# Population attitudes towards research use of health care registries: a population-based survey in Finland

**DOI:** 10.1186/s12910-015-0040-x

**Published:** 2015-07-17

**Authors:** Katariina Eloranta, Anssi Auvinen

**Affiliations:** 1 Medical School, FI-33014 University of Tampere, Tampere, Finland; 2 School of Health Sciences, FI-33014 University of Tampere, Tampere, Finland

**Keywords:** Informed consent, Medical records, Personal health records, Privacy, Research ethics

## Abstract

**Background:**

Register-based research can provide important and valuable contributions to public health research, but involves ethical issues concerning the balance of public health benefits and individual autonomy. This study aimed to describe the opinions of the Finnish public about these issues.

**Methods:**

Mail survey questionnaire sent to a random sample of 1000 Finns.

**Results:**

Participation proportion was 42 %, with 258 women and 160 men. The majority of the participants (61 %) were willing to provide their identifiable health information for research. Almost half of the participants (48 %) would, nevertheless, like to be informed when their information is used. A third (30 %) indicated no need for informed consent in register-based research, a similar proportion felt it should be obtained for every study, and 40 % thought it necessary in some situations, such as studies addressing a sensitive study topic. As for the best policy for obtaining consent, the majority (86 %) favoured broader consent methods: one consent covering a certain register or a research topic. Half of the participants (55 %) desired a required ethical evaluation from register-based research addressing a sensitive issue. Privacy protection was the most common concern for register-based research. More than half of the participants were either content with the current Finnish laws concerning register-based research or wanted to liberalize them to advance research.

**Conclusions:**

The Finnish public is supportive of register-based research, but the requirement for informed consent divides opinions and many would at least like to be informed of the research use of their information.

**Electronic supplementary material:**

The online version of this article (doi:10.1186/s12910-015-0040-x) contains supplementary material, which is available to authorized users.

## Background

Health care registers have been defined as organized systems with uniform data aimed at comprehensive coverage of a target population with a particular disease, condition or exposure [1]. Register-based health research is research based on health care registries or databases, some of which are originally not created for research use [2]. In Finland and other Nordic countries, numerous national health registries have been established with a legislative mandate for public health surveillance, health care monitoring and administrative purposes [3–5]. In this study, we assess common views regarding the justification for research use of health care registries with identifiable personal information, such as hospital discharge registers, cancer registries and medical birth registries. Information in different registries can be linked with a unique identification number given to every citizen and permanent resident in the Nordic countries [3, 5–7]. Our approach is based on the four principles of autonomy, justice, beneficence and non-maleficence [8]. Emphasis is placed on autonomy and the rights of the registrees, versus beneficence and the common good. In this context, the common good derives from the contributions register-based research offers to public health, and is something shared by all or most members of the society.

Register-based research has the potential to offer invaluable benefits for public health and hence, society [7]. The research possibilities of health care registries are extensive. Register-based research can provide important information about the occurrence and aetiology of diseases and the frequency of risk factors, and also enables monitoring their changes over time (surveillance) [3, 9]. A major strength for research is that the data is already available and covers the entire target population [3, 4]. Register-based research can be utilized for assessing the quality and effectiveness of health care, identifying health needs and emerging health issues, designing public health prevention strategies, and allocating limited resources more efficiently [2, 9, 10]. Benefits reach the whole society and thus increase the common good. These benefits can be maximized by following the highest standards of research and efficient utilization of existing registries. Restrictions on research use of registry data would diminish the public health gains from research, reducing the possibilities to develop health care and to respond to new challenges [10].

Despite the obvious benefits, several questions concerning register-based research are debated, and currently also topical, due to the EU Data Protection Directive reform. The key issue is finding the balance between research, with potential public health benefits, and respect for autonomy, with privacy protection and need for informed consent. The pertinent question is whether an individual’s right to privacy is more important than the common good from register-based research, and under what conditions [9–16].

Respect for persons denotes a right to self-determination [9, 17]. In clinical research, informed consent is obtained from the participants, allowing researchers to view their personal data. The strongest requirement for consent applies to intervention studies. This is, however, not directly applicable to register-based research, where the more pertinent issue is to what extent people should have the right to decide who sees their personal information, what details of their lives they want to share, and how their information is being used. In register-based research, obtaining informed consent from thousands of study subjects is time-consuming and financially burdening, or in some cases impossible, since in retrospective studies typically some of the participants are deceased [18]. Obtaining informed consent can also result in selection bias, because of the differences between the consenters and non-consenters, for example by gender, socioeconomic status and health status [10, 18, 19]. The requirement for informed consent is not absolute; the limitations it imposes on research, and therefore public health, must be weighed against enhancement of autonomy [14, 20]. If the study is of importance for public health, the societal benefit may outweigh the potential risk for the study subject, and an individual consent can be waived [9, 14, 15].

The principle of justice entails equality among people and the fair distribution of benefits and burdens [9, 17]. Justice also means equality in health care: everyone’s right to receive adequate treatment [9]. Register-based public health research can provide important information about the quality of the health care system and point out the problem areas [2]. Since we are all potential users of public health care, it can be argued that we have an obligation to provide our information for research to help advance an efficient and functional health care system [21]. On the other hand, research information about the aetiology of diseases and efficacy of treatment enables offering the best treatment based on scientific evidence. This minimizes the adverse effects of diseases and helps a vulnerable group of people, thus promoting equality among the community. Allowing use of one’s information for research can therefore be justified on the basis of solidarity and reciprocity.

The principle of non-maleficence means minimizing potential harm to the study participants [9]. Researchers have an obligation to maintain an appropriate standard of conduct, i.e. follow good research practices, including prior ethical assessment of the benefits and risks. For register-based research, the possible harm comes from the invasion of privacy and misuse of personal information; it differs from clinical medical research in that there is no risk of physical adverse effects [9, 12, 18]. A breach of confidentiality with improper disclosure of sensitive information can lead to discrimination, problems in social and interpersonal relationships and stigmatization [9, 10, 12, 14, 22]. Obtaining permission to conduct research involves a requirement for the researchers to follow prudent practice, including adhering to high quality data protection procedures to ensure confidentiality. With appropriate safeguards, the risk for a breach of confidentiality is minimal [2].

If values are seen as a set of socially constructed norms, their assessment of acceptability needs to be conducted in a specific social context, as perception of what is justified and what is not depends on culture, population and time. Therefore, the systematic exploration of the diversity of views to describe ‘common sense of justice’ (sometimes called ‘common morality’) is a worthwhile research goal. Commonly held values or views of a specific issue can be regarded as prescriptive, forming the basis of a code of conduct and justifying institutionalized obligations such as directives, laws and other regulations. In a democracy, legislation needs to be coherent with the prevailing views of what is fair and acceptable, making it imperative to explore public opinion [20].

Legislation regarding the use of health records and national registries for research varies between countries. From the researchers’ point of view, the legislation in Finland and other Nordic countries is more liberal than in many other European countries, and enables extensive public health research with a reasonably expedient process for obtaining research permission [23]. The Finnish legislation allows register-based research without informed consent, provided that the study is solely based on registers (involving no contact with the study subjects), the study is considered to be of public health importance, and the number of study participants is large [23]. In some other European countries, the protection of privacy and individual rights are considered extremely important and the legislation is stricter. Therefore, Finland provides a case study of public acceptance of a liberal model and whether there is demands for stricter limits for research use of registry data.

The 1995 EU Data Protection Directive sets the rules for strict protection of personal data, but the implementation of these rules differs between the Member States [24, 25]. Currently, the EU Data Protection Directive is being revised, aiming to harmonize data protection rules across the EU and tighten privacy protection of personal information [24, 26]. Amendments proposed by the European Parliament Committee on Civil Liberties, Justice and Home Affairs include, for example, obligatory pseudonymisation of personal data and the requirement of explicit consent when using personal health data for scientific purposes [27]. Considering the latter, there is an accompanying amendment that allows Member States to make exceptions with high public interest research, which can then again lead to different practices among European countries [27, 28]. If these amendments come to effect, they may in the future lead to substantial impediments for public health research [25, 26, 28].

Earlier studies on this subject have found that generally the support for health research is high. [29–31] Privacy protection is a common concern, and the risks involving research use of personal data are recognized. [19, 29, 31–33] The results have, nevertheless, shown the public has been willing to allow the use of their information and to consider alternative methods for study-by-study consent. [30, 34–36] The aim of this study was to describe the opinions of the Finnish people about the ethical issues in health research based on register data.

## Methods

This study was conducted as a mail survey. A questionnaire was developed on the basis of previous studies and the current regulation in Finland, as well as other countries, and with also the proposed revision to the EU Directive in mind. Some of the questions were phrased to match with those of previous questionnaires to allow a comparison of results. Pilot testing for comprehensibility and clarity was conducted with a small group (5 volunteers). According to Finnish regulations, no ethical committee review is required for questionnaire surveys, and such was not conducted for this study. The questionnaire was sent to a random sample of 1000 Finns (identified by the Finnish Population Register Centre) (542 women and 458 men), with a letter containing information about this study and a prepaid return envelope. Inclusion criteria were the age of 18–80 years and Finnish as their first language. Three weeks after the first letters were sent, reminders were sent to those who had not yet responded (using a tracking number printed in the return envelopes).

Most of the 44 items in the questionnaire were multiple choice questions, with a few complementary open questions (see Additional file 1). The questionnaire covered demographic information (such as age, gender, and education level), health status and use of health care services, previous participation in health research, general attitude towards medical and public health research, and opinions about the ethical questions involving register-based health studies. The questions mapped the attitudes towards informing study participants about the research use of their information, need for informed consent, legislation regarding register-based research, need for ethics committee reviews and reporting of register-based public health studies. Due to the unfamiliarity of the study theme information about health research, register-based research and the ethical problems, legislation and current practises involving them was also provided in the questionnaire form placed strategically in between the questions (see Additional file 1).

The questionnaire data were analysed using descriptive statistics, such as frequencies, and statistical significance was assessed using chi-square tests (Pearson chi-square and linear-by-linear association). Education level was classified as: 1: primary school or lower basic education, 2: secondary school or higher basic education, 3: high school graduate or vocational degree, 4: University of Applied Sciences degree or Bachelors degree (corresponding to a college degree), 5: Master’s degree or higher.

## Results

A total of 423 questionnaires were returned; participation was 42 % (among women 48 % and men 36 %). Five participants were excluded due to insufficient data (more than 50 % missing). Of the subjects included in the analysis, 258 were women (62 %) and 160 men (38 %). The age group 61–70 years old had the highest participation proportion (65 %) and made up 30 % of the participants (Table 1). Women were a majority in every age group (Table 1).

**Table 1:** Characteristics of the study population

Age (years)	n (%)	Women n (%)	Men n (%)
<=30	44 (10.5)	28 (63.6)	16 (36.3)
31-40	47 (11.2)	31 (66.0)	16 (34.0)
41-50	57 (13.6)	30 (52.6)	27 (47.4)
51-60	88 (21.1)	62 (70.5)	26 (29.5)
61-70	126 (30.1)	75 (59.5)	51 (40.5)
>70	49 (11.7)	26 (53.1)	23 (46.9)
Missing	7 (1.7)	6 (2.3)	1 (0.6)
Education	n (%)	Women n (%)	Men n (%)
Primary school	74 (17.7)	36 (48.6)	38 (51.4)
Secondary school	44 (10.5)	31 (70.5)	13 (29.5)
High school	165 (39.5)	105 (63.6)	60 (36.4)
University of Applied Sciences or Bachelor’s degree	80 (19.1)	52 (65.0)	28 (35.0)
Master’s degree or higher	52 (12.4)	33 (63.5)	19 (36.5)
Missing	3 (0.7)	1 (0.4)	2 (1.3)
Total	418 (100.0)	258 (61.7)	160 (38.3)

Of the participants, 40 % had high school and 32 % university level education. The majority of participants with higher education were women (level 4: 65 % and level 5: 64 %) (Table 1). In terms of health status, 117 (28 %) of the participants reported poor health (a long-term illness and a self-reported health status of moderate to very poor) and 164 (39 %) participants indicated good health (no long-term illnesses and a self-reported health status of fairly good to very good). Missing item response was on average 2.0 %, range 0–7.2 %. The remaining participants could not be placed in either category (i.e. reported, for example, poor health but had no long-term illnesses).

Most of the participants (83 %) had a positive or very positive opinion about health research in general (on a five-point scale from very negative to very positive), and register-based research was considered to be an important or very important part of it by 88 % (on a four-point scale from not at all important to very important). The majority also had a positive (49 %) or very positive (19 %) opinion about using administrative health registries for research purposes (on a five-point scale). Of the remaining, more than a quarter (27 %) had a neutral attitude, while only 3 % had a negative or very negative opinion. Commonly supported research purposes for health registries included aetiologic studies 76 %, disease monitoring 60 %, assessing the effectiveness of health care 53 %, and any research use 38 % (multiple-choice question). Of the study participants, 16 % were aware of at least one Finnish register-based study. The current practice of informing about new/ongoing register-based studies was seen as inadequate by 45 %, with almost half the participants uncertain (49 % answered “Do not know”). Only 6 % felt that information about ongoing register-based research was sufficiently available.

**Fig. 1:**
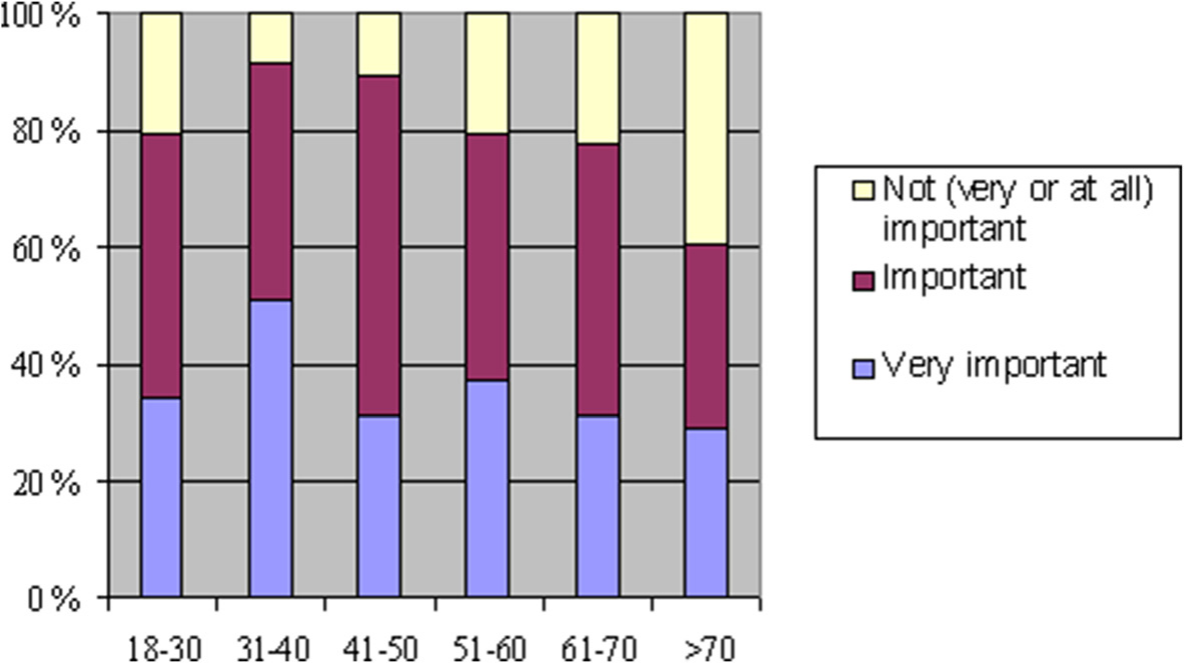
The importance of privacy protection of health information in different age groups

The majority of participants had a positive (48 %) or very positive (12 %) attitude towards the use of their own health information in register-based research, with 28 % having a neutral attitude, 9 % a negative and only 1 % a very negative attitude. Those with a higher education were more often positively inclined than those with a lower education level: of the participants with the highest education level (level 5), 82 % had a positive or very positive opinion, while of the participants with the lowest education level, only 41 % (p < 0.001). Gender, age or health status did not affect the results. Privacy protection of health information was regarded as very important by 36 % and important by 44 %. Of those respondents, 11 % had a very positive and 45 % positive attitude towards their own information being used for research, with 31 % having a neutral, 11 % a negative and 2 % a very negative view. The results are very similar to those of the whole sample population. A smaller proportion thought privacy protection as not very important (19 %) or not at all important (1 %). The oldest age group (>70 years) was less concerned with privacy than the younger ones: in the age group 31–40 years 92% held privacy of their health information as very important or important, while in the oldest age group the percentage was only 61 % (p = 0.007, Fig. 1). There was, however, no linear association. Gender, education or health status did not affect the view significantly. Of the participants, 45 % somewhat agreed and 7 % strongly agreed with the statement “A register-based study of major public health impact is more important than privacy protection of an individual person”. A quarter (25 %) somewhat disagreed and 12 % strongly disagreed. Health status was a significant factor: the participants with good health were more likely to somewhat or strongly agree (56 %) than the participants with poor health (40 %) (p = 0.04).

Almost half of the participants (48 %) would like to be informed every time their information is being used for register-based research (Table 2). A third (33 %) thought that in some cases informing is not necessary, and 19 % found informing altogether unnecessary (Tables 2 and 3). Participants with poor health were more likely to regard informing always necessary than the participants with good health (55 % vs. 43 %) (p = 0.034). There was no linear association with age and the need to be informed; however, the youngest (18–30 years) and the oldest (>70 years) age group found informing unnecessary more often than the other age groups (p = 0.028, Table 2). The level of education also had some influence: the participants with a university education (level 4 or 5) were more often of the opinion that in some cases informing is not necessary than the participants with basic education (level 1 or 2), (p = 0.008), but no linear association was found there, either. Gender did not affect the results (Table 2).

**Table 2:** Views of the study population on informing and informed consent

Would you like to be informed if your information would be used for research purposes?	Women %	Men %	<=30 %	31-40 %	41-50 %	51-60 %	61-70 %	>70 %	Total %
I would like to be informed every time	47.5	48.1	31.8	48.9	56.1	48.3	56.0	29.2	47.8
In some cases there is no need to inform	35.3	30.0	36.4	36.2	24.6	36.8	28.0	43.8	33.1
No need to inform at all	17.3	21.9	31.8	14.9	19.3	14.9	16.0	27.1	19.1
			p = 0.028						
Should an informed consent be obtained from every study participant in a register-based study?	Women %	Men %	<=30 %	31-40 %	41-50 %	51-60 %	61-70 %	>70 %	Total %
No need for obtaining informed consent	26.5	37.1	29.5	23.4	33.3	23.9	35.2	33.3	30.3
In some cases informed consent should be obtained	44.4	31.4	50.0	44.7	36.8	43.2	35.2	33.3	39.6
Informed consent should always be obtained	29.2	31.4	20.5	31.9	29.8	33.0	29.6	33.3	30.1
	p = 0.019								
What kind of practice would be best, if informed consent was required for register-based studies?	Women %	Men %	<=30 %	31-40 %	41-50 %	51-60 %	61-70 %	>70 %	Total %
Informed consent should be obtained for every study	12.9	13.5	9.1	13.3	14.3	8.1	17.2	15.9	13.4
One informed consent for one field of research (for example cancer research)	43.3	47.1	50.0	44.4	41.4	46.5	45.9	36.4	44.6
One informed consent for research use of a certain register	43.4	38.1	40.9	42.2	41.4	45.9	36.9	45.5	41.3
Other	0.4	1.3	0.0	0.0	3.6	0.0	0.0	2.3	0.8

**Table 3:** Special situations for informing and informed consent

In what kind of cases there is no need to inform about the research use of your information?	Women %	Men %	Total %
If the research topic is of public health importance	52.8	35.4	46.4
If the study is a continuation to a previous study of which you have already been informed about	57.3	58.3	57.2
If a government research centre, a university, or some other reliable organization is in charge of the study	52.8	43.8	49.3
If the amount of study participants is so high that informing all of them is extremely difficult	56.2	45.8	52.2
Other	4.5	2.1	3.6
In what kind of cases would you like to be asked for your personal consent for participation?	Women %	Men %	Total %
If the research topic or the information used for it are sensitive	74.6	68.0	72.6
If the research results might stigmatize a group of people	55.3	44.0	51.8
If the practical applicability of the study is unclear	38.6	34.0	37.2
If the research topic is not of public health importance	36.0	40.0	37.2
Other	2.6	4.0	3.0

Most of the participants (75 %) would like to be informed of the possible research use of their medical records when being admitted to the hospital. Half of the participants (52 %) would like to be able to limit research use of their medical records and 11 % to forbid it altogether. Still, more than a quarter (29 %) thought that everyone’s medical records should be accessible for research use. The results were unaffected by health status. We also asked about some well-known Finnish administrative health registries and whether the participants would like to be able to limit or forbid the research use of the information they contain (Table 4).

**Table 4:** Opinions on the research use of information in different registers

Would you like to be able to limit or forbid the research use of the information contained in the following national registers?				
	Yes, forbid the use altogether	Yes, limit the use of some information	No, everyone’s information should be accessible for researchers	Do not know
Cancer register	8 %	27 %	48 %	11 %
Medical birth register	9 %	30 %	43 %	14 %
Care register for Health Care (HILMO)	11 %	38 %	38 %	11 %

As for register linkage, almost a third of the participants (32 %) would like to be able to limit linkage of certain registries, but a slightly larger proportion (34 %) felt that everyone’s information should be available for researchers in every national register. Only a minority (15 %) would forbid record linkage altogether.

The requirement for informed consent divided the participants into three groups of similar size (Table 2). A little less than a third (30 %) thought that there is no need in any case to obtain informed consent from the study participants in register-based studies, while the same proportion (30 %) found it should be obtained every time, and a slightly larger proportion (39 %) thought that it should be obtained in some special cases (Table 3). Men found informed consent unnecessary more often than women (37 % vs. 27 %), whereas women more frequently thought that in special cases informed consent should be required (44 % vs. 31 %) (p = 0.019, Table 2). Participants with the highest education (level 5) were less likely to always require an informed consent than those with the lowest educational level (15 % vs. 42 %; p = 0.002). Age or health status did not have a statistically significant effect on these results.

When asked more specifically about the best policy for obtaining informed consent, if it was required for register-based studies in Finland, 45 % suggested a single informed consent for a certain research topic (for example cancer research) (Table 2). Nearly as many (41 %) supported single informed consent for the research use of a certain register, and only a minority (13 %) endorsed a study-by-study approach (Table 2). Of the participants in good health, more than half (51 %) selected the register-specific option, 36 % the topic-specific alternative and 12 % the study-by-study method, while of the participants in poor health, the register-specific alternative was chosen by only a third (34 %), the topic-specific by 46 % and the study-by-study method by 19 % (p = 0.037). Gender, age or education level did not influence the results significantly.

Almost a third of the study population (29 %) thought that the differences in legislation concerning register-based research among European countries are not a problem. Still, 40 % of the participants preferred legislation to be someway harmonised in Europe, 20 % found that legislation should be changed in other countries closer to the Nordic model, and 9 % indicated that the Nordic countries should shift towards the policy of other European countries. When asked about Finnish legislation, 35 % of the study population wanted to tighten the law for some parts, 28 % were satisfied with the current practices, and 25 % wanted to liberalize the law to further advance scientific research.

More than half the participants (55 %) would like an ethics committee to evaluate only those register-based studies addressing a sensitive topic. A quarter (25 %) would require ethical evaluation of all register-based studies, and the rest (20 %) thought that the current Finnish practice requiring no ethical evaluations is satisfactory. If an ethical evaluation for a register-based study had been conducted, 18 % of the participants would no longer require an informed consent, but 48 % felt that consent would still be needed.

**Fig. 2:**
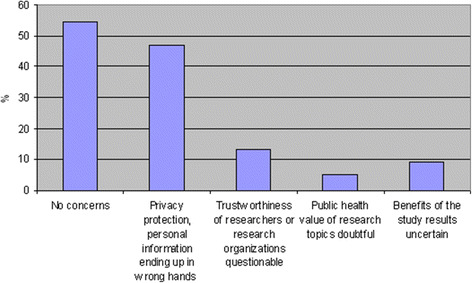
Concerns of participants regarding register-based research

Finally, the participants were asked about concerns regarding register-based research. More than half (55 %) indicated no concerns, but many of the participants were worried about privacy protection (47 %) (multiple-choice question) (Fig. 2).

Women and older subjects participated more actively in this study. We analysed the results with correction for gender and age distributions to correspond to the Finnish population. The results showed only one significant association with gender, which was on the need to obtain informed consent, where men found informed consent unnecessary more often. Corrections had only minor influence on the results: no need for informed consent 30 % uncorrected vs. 31 % corrected, 39 % vs. 38 % in some cases, and no change in the proportion who would require informed consent every time. Correction for age also only slightly affected the results. The oldest age group was less concerned with privacy of their health information than younger age groups. For the importance of privacy protection the age-corrected results were: no change in those who regarded it as very important, 44 % uncorrected vs. 45 % corrected regarded as important, 19 % vs.18 % not very important. The oldest and the youngest age groups considered informing about the research use of personal information unnecessary more often than the other age groups. After correction, 47 % wanted to be informed always, 33 % in some cases and 20 % never. The figures were very similar to the original results. Also, the differences in results between those participants who returned the questionnaire early and those who answered late (after the reminder letter) were minor. Of the early responders, 62 % and of the late responders, 61 % had a positive view on using their health information for research. For informed consent, among the early responders 31 % no need vs. 23 % among late responders, 39 % vs. 44 % in some cases and 30 % vs. 33 % always.

## Discussion

Our findings can contribute to moral justification conducted as reasoning through reflection, seeking coherence for various viewpoints: describing and elaborating multiple and possibly contradictory interpretations representing various views or beliefs in a pragmatic fashion. Reflective equilibrium as a matter of interpretation of a specific issue needs to fully incorporate various views or judgements. Descriptive ethics provides a method for compiling the views and interpretations of people involved as stakeholders and to identify views that need to be accommodated in further elaboration, in order for further arguments to cover an adequate range of interpretations.

Based on the views expressed in a survey such as ours, a compromise can be obtained, which is acceptable from all expressed points of view. We believe developing a reflective equilibrium requires interplay of actual judgements of specific issues and moral theory or normative considerations. We propose that the views, perceptions and interpretations should guide rules and regulations, which in turn guide practice, such as register-based health research. This can be seen as a variant of autonomy, an indirect influence by citizens that are unaware of their future position in the practice, i.e. whether they will be users of health care services and actual stakeholders as patient registrees.

This study showed that the Finnish public has a positive opinion about health research in general and also sees register-based research as an important part of it. Scientific research is considered important for public health, and most of the participants regarded the research use of national registries as positive. The majority of the participants (80 %) considered privacy protection of their health information important or very important. Of note, 56 % of those respondents had nevertheless positive or very positive attitude about their information be used for research purposes, with 31 % being of neutral point of view. This suggests that the respondents trust the research organizations and their data protection practices. The Finnish legislation concerning register-based research raised no alarm. More than 50 % indicated that the current laws are satisfactory, or that they could even be more liberal. If the EU Data Protection Directive comes to form with the amendments currently planned by the European Parliament, the Finnish practices would have to change quite dramatically, with more restrictions for register-based research. This study indicates that the Finnish public does not fully agree with the need for this.

The views on the importance of both privacy and public health research found in this study were consistent with previous studies [29–32, 34, 36]. In our study, the majority had a positive view on both the research use of national registries (68 %) and of their own identifiable information (60 %). In Great Britain, a national survey studying the public’s views on the use of identifiable data for health research purposes by the National Cancer Registry found that the majority (72 %) of the respondents did not see it as an invasion of privacy [34]. Similar findings were obtained in a telephone survey in Australia concerning the research use of identifiable data by the Western Australia Birth Defects Registry [36]. In our study privacy protection was regarded as important or very important by 80 % and was the most common concern (47 %). In a Canadian telephone survey concerning public opinions about consent to access personal information for health research, privacy protection was regarded as somewhat important by 23 % and very important by 74 % [30]. An Australian mix-method study, examining the public views on the privacy protection of their health information, showed that medical research was widely supported (98 %), but privacy concerns were common (66 %) [31]. Also, the results from focus group studies in Ireland and Great Britain about the research use of general practice medical records or primary care patient record data showed that although health research was generally supported, privacy protection raised concern [29, 32]. The Canadian telephone survey included a similar statement as in our study: “Research that could be beneficial to people’s health is more important than protecting people’s privacy”. The results somewhat differed from ours with 31 % of the participants strongly agreeing in the Canadian survey, while only 7 % strongly agreeing in our material [30]. The proportions somewhat agreeing were quite similar (37 % in the Canadian study vs. 45 % in our study), and the response options were the same in the two studies (strongly agree, somewhat agree, somewhat disagree, strongly disagree and do not know).

Informing about the research use of one’s register information was regarded as important in our study: half of the participants wanted to be informed in every instance and a third at least in some cases. Under the current law in Finland, the study subjects need not be informed of the research use of their register information, presenting a contradiction with the public views. Less than a third (30 %) thought that informed consent should always be required for register-based research; a common view was that it should be left for special cases, and another 30 % felt it was not needed at all. When the policy for obtaining informed consent was specified, only a minority felt it necessary to be obtained for every project, while the majority opted for broader consent methods (a consent covering the research use in a certain field of research or for a certain register). The Finnish public seems to be satisfied with just being informed what their personal data is being used for, even though they are not asked for permission. This differs from the Australian mix-method study, where the majority (92 %) wanted to be asked for permission before the use their information for other purposes than medical treatment [31]. Also, in a Canadian survey on patient opinions about consent and research use of medical records, 78 % felt the need for a consent for the use of identifiable data and 70 % wanted new consent for each project [37]. In an Irish mixed-methods study, 72 % preferred to be asked before their identifiable information was passed on [35]. In a Japanese focus group study about the attitudes of both the public and medical doctors towards the research use of data and samples without informed consent, the results indicated that the public has divergent attitudes about research use of information without consent, and that the opinions of doctors and lay public differ greatly [33]. Our results are, however, consistent with the Canadian telephone survey, where only 32 % wanted to be asked for permission every time, 28 % were willing to give their general permission and 24 % chose a notification with a possibility to opt out [30].

The study population considered ethics committee evaluations important for studies on a sensitive subject. Currently in Finland, ethical evaluations are only required from medical studies that involve a risk of physical harm, but not for register-based research. This is contrary to the practices in many other countries, where ethical evaluation is required also for register-based research, to consider the need for informed consent, ensure adequate data safety and weigh the potential risks for the subjects against the benefits. However, as the risk to the participants in register-based studies is usually minimal, the compulsory ethical evaluation can cause unnecessary impediments for research. The procedure may cause delays and the requirement for more extensive evaluation also increases overall expenses. On the other hand, different national practices create inequality if a similar study can be allowed in one country and denied in another.

In this study, concern about privacy was more common among younger people, yet they found informing about the research use of their personal information unnecessary more often, and age did not affect the view on the need of informed consent. In other words, even the younger participants, despite their higher concern for privacy, trust researchers and are willing to let their information being used for research. Gender affected the results very little, only the need for an informed consent was indicated less frequently by men than women. The education level influenced the results to some extent: those with a higher education were very supportive of research, had a more positive opinion about their own information being used for research, more often found that informing about research use of personal information is not always necessary and thought that informed consent should not be required from every register-based study. A higher educational level has been linked with having a more positive attitude towards scientific research [38]. This might be due to being more familiar with research and different research methods, reflecting also a deeper understanding of the benefits of research. Health status did not affect the view on the research use of medical records or the opinion of the importance of privacy protection; this was consistent with the Australian mix-method study [31].

Of the respondents, 45 % indicated that the current methods for informing about on-going and new register-based studies in Finland was inadequate. Most of the respondents felt they were unable to obtain sufficient information about register-based studies, even if they tried. A large proportion (49 %) did not know whether the informing is adequate or not, perhaps because the whole concept of register-based research is largely unknown to the general public, and even the little information that is available has gone unnoticed.

The study participants were largely representative of the Finnish population. The education levels of the participants corresponded to the education levels of the Finnish population [39]. There were, however, more women and more participants in the older age groups than in the general population [40]. Correction for age and gender distribution to match the entire population yielded almost unaltered results. Hence, it appears that selection bias did not materially affect the results.

Most of the open comments at the end of the questionnaire related to the difficulty to respond because register-based research as a topic is so unfamiliar. Although we provided basic information on the subject, the survey questionnaire did not allow detailed coverage of the different uses and regulations of register data. Some of the answers might have been different with a better knowledge and understanding of the topic. This could have been better achieved with an alternative approach, such as focus group or in-person interview, when also a more extensive elaboration on the respondents own views would have been possible. Another limitation in this study was that the questionnaire was not validated or entirely consistent with those of previous studies. However, due to differences in legislation and practices, a completely uniform questionnaire would likely not be feasible.

## Conclusions

The Finnish people are generally very supportive of health research and regard register-based research as important and necessary. The general opinion about the research use of national health registries is positive, and people are willing to provide their own information for research use. Informed consent in register-based studies divides opinions, but overall, the majority feels that it is needed only in special cases or not at all, and support alternative consent methods. The majority would still like to be informed when their personal information is being used for research purposes. Ethics committee reviews were found useful, but only a minority felt they should be required routinely from every register-based study. The current Finnish legislation gained support from the majority of participants; many were even inclined to liberalize it for the benefit of research. When it comes to register-based research, the Finnish public seems to hold benefit for public health as more important than the individual right to privacy and indicate no acute need for restrictions for register-based research. These results demonstrate that the value of register research is widely recognized among the public and a common altruistic attitude allows its use.

## Additional file

Additional file 1: **Survey questions and related information translated to English.** The file provides the questionnaire translated to English, edited in the same way as in the original questionnaire in Finnish.

## References

[1] Gliklich R, Dreyer R, Leavy M, editors. Registries for Evaluating Patient Outcomes: A User’s Guide. 3rd ed. Agency for Healthcare Research and Quality. 2014. Publication No. 13(14)-EHC111. http://www.effectivehealthcare.ahrq.gov/registries-guide-3.cfm. Accessed 1 Apr 2015. 24945055

[2] Rosén M, Hakulinen T, Wolfgang A, Pigeot I (2005). Use of Disease Registers. Handbook of epidemiology.

[3] Sorensen HT (1997). Regional administrative health registries as a resource in clinical epidemiology: A study of options, strengths, limitations and data quality provided with examples of use. Int J Risk Saf Med.

[4] Thygesen LC, Ersboll AK (2014). When the entire population is the sample: strengths and limitations in register-based epidemiology. Eur J Epidemiol.

[5] Rosen M (1999). Data needs in studies on equity in health and access to care--ethical considerations. Acta Oncol.

[6] Ludvigsson JF, Otterblad-Olausson P, Pettersson BU, Ekbom A (2009). The Swedish personal identity number: possibilities and pitfalls in healthcare and medical research. Eur J Epidemiol.

[7] Olsen J (2011). Register-based research: some methodological considerations. Scand J Public Health.

[8] Beauchamp TL, Childress JF (2001). Principles of Biomedical Ethics.

[9] Coughlin SS (2006). Ethical issues in epidemiologic research and public health practice. Emerg Themes Epidemiol.

[10] Gostin LO (2001). Health information: reconciling personal privacy with the public good of human health. Health Care Anal.

[11] Fairchild AL, Johns DM (2012). Beyond bioethics: reckoning with the public health paradigm. Am J Public Health.

[12] Coughlin SS, Beauchamp TL, Weed DL. Ethics and epidemiology. 2nd. ed. New York: Oxford University Press; cop. 2009.

[13] Coughlin SS (2000). Ethics in epidemiology at the end of the 20th century: ethics, values, and mission statements. Epidemiol Rev.

[14] Regidor E (2004). The use of personal data from medical records and biological materials: ethical perspectives and the basis for legal restrictions in health research. Soc Sci Med.

[15] Thurston WE, Burgess MM, Adair CE (1999). Commentary: ethical issues in the use of computerized databases for epidemiologic and other health research. Chronic Dis Can.

[16] Etzioni A (2011). On a communitarian approach to bioethics. Theor Med Bioeth.

[17] Dawson A (2011). Public Health Ethics: Key Concepts and Issues in Policy and Practice.

[18] Horner JS (1998). Research, ethics and privacy: the limits of knowledge. Public Health.

[19] Hill EM, Turner EL, Martin RM, Donovan JL (2013). "Let's get the best quality research we can": public awareness and acceptance of consent to use existing data in health research: a systematic review and qualitative study. BMC Med Res Methodol.

[20] Childress JF, Faden RR, Gaare RD, Gostin LO, Kahn J, Bonnie RJ (2002). Public health ethics: mapping the terrain. J Law Med Ethics.

[21] Miller FG (2008). Research on medical records without informed consent. J Law Med Ethics.

[22] Marshall PA, Rotimi C (2001). Ethical Challenges in Community-Based Research. Am J Med Sci.

[23] Lehtonen LA (2002). Government registries containing sensitive health data and the implementation of EU directive on the protection of personal data in Finland. Med Law.

[24] European Commision: Commision proposes a comprehensive reform of the data protection rules. 2013. http://ec.europa.eu/justice/newsroom/data-protection/news/120125_en.htm. Accessed 3 Dec 2013.

[25] Andersen MR, Storm HH, Eurocourse Work Package 2 Group (2015). Cancer registration, public health and the reform of the European data protection framework: Abandoning or improving European public health research?. Eur J Cancer.

[26] Fears R, Brand H, Frackowiak R, Pastoret PP, Souhami R, Thompson B (2014). Data protection regulation and the promotion of health research: getting the balance right. QJM.

[27] Nyren O, Stenbeck M, Gronberg H (2014). The European Parliament proposal for the new EU General Data Protection Regulation may severely restrict European epidemiological research. Eur J Epidemiol.

[28] Di Iorio CT, Carinci F, Oderkirk J. Health research and systems´ governance are at risk: should the right to data protection override health? J Med Ethics 2013 05.12.2013;0:1–5. doi:10.1136/medethics-2013-101603. 10.1136/medethics-2013-101603 24310171

[29] Clerkin P, Buckley BS, Murphy AW, MacFarlane AE (2013). Patients' views about the use of their personal information from general practice medical records in health research: a qualitative study in Ireland. Fam Pract.

[30] Willison DJ, Schwartz L, Abelson J, Charles C, Swinton M, Northrup D (2007). Alternatives to project-specific consent for access to personal information for health research: what is the opinion of the Canadian public?. J Am Med Inform Assoc.

[31] King T, Brankovic L, Gillard P (2012). Perspectives of Australian adults about protecting the privacy of their health information in statistical databases. Int J Med Inf.

[32] Robling MR, Hood K, Houston H, Pill R, Fay J, Evans HM (2004). Public attitudes towards the use of primary care patient record data in medical research without consent: a qualitative study. J Med Ethics.

[33] Asai A, Ohnishi M, Nishigaki E, Sekimoto M, Fukuhara S, Fukui T (2002). Attitudes of the Japanese public and doctors towards use of archived information and samples without informed consent: preliminary findings based on focus group interviews. BMC Med Ethics.

[34] Barrett G, Cassell JA, Peacock JL, Coleman MP (2006). National survey of British public's views on use of identifiable medical data by the National Cancer Registry. BMJ.

[35] Buckley BS, Murphy AW, MacFarlane AE (2011). Public attitudes to the use in research of personal health information from general practitioners' records: a survey of the Irish general public. J Med Ethics.

[36] Molster C, Bower C, O'Leary P (2007). Community attitudes to the collection and use of identifiable data for health research--is it an invasion of privacy?. Aust N Z J Public Health.

[37] Page SA, Mitchell I (2006). Patients' opinions on privacy, consent and the disclosure of health information for medical research. Chronic Dis Can.

[38] Bak H (2001). Education and Public Attitudes toward Science: Implications for the "Deficit Model" of Education and Support for Science and Technology. Soc Sci Q.

[39] Official Statistics of Finland (OSF): Educational structure of population. ISSN = 2242-2919. Appendix table 1. Population aged 15 or over by level of education and gender 2012 . Helsinki: Statistics Finland. 2013. http://www.stat.fi/til/vkour/2012/vkour_2012_2013-12-04_tau_001_en.html. Accessed 4 Dec 2013.

[40] Official Statistics of Finland (OSF): Population structure.ISSN = 1797-5395. Helsinki: Statistics Finland. 2013. http://www.tilastokeskus.fi/til/vaerak/index_en.html. Accessed 2 Mar 2014.

