# Supplementary material for: Population attitudes towards research use of health care registries: a population-based survey in Finland
Source: BMC Med Ethics. 2015 Jul 17;16:48. doi: 10.1186/s12910-015-0040-x (PMC4504396; doi:10.1186/s12910-015-0040-x)
Supplement: Supplementary file 1 — Survey questions and related information translated to English. The file provides the questionnaire translated to English, edited in the same way as in the original questionnaire in Finnish. [file 12910_2015_40_MOESM1_ESM.pdf]

Population attitudes towards research use of health care registers

Survey questions and related information translated to English

Basic information

- Sex
  1 Female
  2 Male

- Age ______ years

- What is your highest educational degree?
  1 Lower basic level or primary school
  2 Higher basic level or secondary school
  3 High school degree, vocational degree
  4 University of Applied Sciences degree or Bachelor's degree
  5 Master's degree
  6 Doctor of Philosophy (PhD)

- What is your opinion of your health condition compared to your peers?
  1 Very good
  2 Fairly good
  3 Moderate
  4 Poor
  5 Very poor

- Do you have a long-term medical illness requiring (almost) constant medication?
  1 No
  2 Yes. Which illness? ______________________________________

  - Does your illness affect your daily life in a negative way?
    1 Yes, significantly
    2 Yes, somewhat
    3 No, mostly not
    4 No, not at all

- Have you seen a doctor due to an illness or an accident during the last year?
  1 No
  2 Yes. How many times? ______

- Have been admitted to hospital due to an illness during the last three years?
    1 No
    2 Yes. How many times? ______

Previous experience from health research

Medical and public health research are used for example mapping the state of public health, the prevalence and etiology of diseases, assessing the effectiveness of health care. In health research the research subject is a group of people, a study population. The size of the study population can vary from a few dozen to several million people. There are several types of studies. Here we focus on three types of health research, two which we define below. The third type is register-based research, which we will focus on later.

Clinical intervention study: A study focusing on for example an effect of a new drug compared to existing medicines. In place of a drug, other studies can explore the efficacy of a certain treatment procedure, for example an operation. The study participants are usually randomized in an intervention group and a control group.

Survey or in-person interview study: The data for the study is gathered with in-person interviews with the study participants or with questionnaire forms. The study can be a follow-up study lasting several years or decades. These studies are used for example for finding out the risk factors and etiology of diseases and the effects on quality of life.

- Have you ever participated in a medical or public health study?
    1 No
    2 Yes

    - In what kind of medical or public health study you have participated?
        1 Clinical intervention study
        2 Survey
        3 Other. What? ______________________________________

    - What kind of experience it was for you to participate?
        1 Mostly or altogether positive
        2 Neutral
        3 Mostly or altogether negative

    - Why was your experience mostly or altogether negative?
        1 I experienced harms and/or adverse effects from the study
        2 I received no benefits from the study
        3 Other. What? ____________________________________

- Would you participate again in a medical or public health research?
    1 No
    2 Yes
    3 Do not know

- What is your opinion about medical and public health research in general?
    1 Very positive
    2 Positive
    3 Neutral
    4 Negative
    5 Very negative

- How do you feel about different types if health research?

	Very positive		Neutral		Very negative
Clinical intervention study	1	2	3	4	5
Survey study	1	2	3	4	5

- How important do you find different types of research?

	Very positive		Neutral		Very negative
Clinical intervention study	1	2	3	4	5
Survey study	1	2	3	4	5

- How willing would you be to participate in different types of health research?

	Very positive		Neutral		Very negative
Clinical intervention study	1	2	3	4	5
Survey study	1	2	3	4	5

- What matters do you consider important when participating in a medical or public health study? What are the reasons for participating?

1 Possibility to receive better treatments for myself
2 Possibility for better treatments for future generations
3 Common good, duty to participate
4 Supporting scientific research
5 Participating is easy and effortless
6 Other. What? ___________________________________
7 I do not see any reason to participate in a medical or public health study

Register-based research in general

Register-based research is scientific research based on national registries, many of which are held by government organizations. The registries utilized for health research are often related to the functions of health care organizations and KELA (a Finnish Social Insurance Institution). Examples include cancer register, KELA medical reimbursement register and care register for health care. Register-based health research provides information on public health and prevalence and etiology of diseases. The knowledge gathered from the studies can be used for preventive public health interventions and for assessing the effectiveness of the health care system.

- Do you know a register-based study conducted in Finland?

1 No
2 Yes

- Have you read about the results of a register-based study?

1 No
2 Yes

- Is the public, in your opinion, sufficiently informed about new/on-going register-based studies?
  1 No
  2 Yes
  3 Do not know

- Do you find enough information about new/on-going register-based studies?
  1 No
  2 Yes
  3 Do not know

- What would be the best way to inform about new register-based studies?
  1 Newspaper and/or other mass media
  2 The website of the research organization
  3 The website of the organization maintaining the register
  4 The website for the researchers conducting register-based studies
  5 Other. What? ____

The research use of health registers

- What is your opinion about using administrative health registers for research purposes?
  1 Very positive
  2 Positive
  3 Neutral
  4 Negative
  5 Very negative

- For what kind of research purposes should these national registers be used for?
  1 Any kind of medical or public health research
  2 Monitoring the prevalence of diseases and the changes in public health
  3 Finding out the etiology of diseases
  4 Assessing the effectiveness of treatment
  5 They should not be used for any kind of research
  6 Other. What? ____

- How important do you consider register-based research as a part of medical and public health research?
  1 Very important
  2 Important
  3 Not very important
  4 Not at all important

Privacy protection

Every individual has a right to privacy and that includes a right to keep personal information from outsiders. In Finland the research use of personal data is being controlled by personal data act (22.4.1999/523 www.finlex.fi). The aim of the personal data act is to ensure privacy protection and high standard when processing personal data.

- What is your opinion of using your (identifiable) health information for register-based research?
  1 Very positive
  2 Positive
  3 Neutral
  4 Negative
  5 Very negative

- How important do you consider the privacy protection of your health information?
  1 Very important
  2 Important
  3 Not very important
  4 Not at all important

- Do you agree with the statement: "A register-based study of great public health impact is more important than privacy protection of an individual person"?
  1 Strongly agree
  2 Somewhat agree
  3 Somewhat disagree
  4 Strongly disagree
  5 Do not know

Informing and informed consent

Under the current law in Finland the study subjects need not be informed when their personal data is being used for research purposes, when the study is based solely on register information and the number of the study subjects is high.

- When using health care services or buying prescription drugs from the pharmacy, personal data is being recorded in different registers. Would you like to be informed about the possible research use of register data in these situations?
  1 Yes
  2 Yes, if the data being registered is of a sensitive nature
  3 No
  4 Do not know

- Would you like to be informed if your health information contained in registers was used for research purposes?
  1 Yes, always
  2 No need to inform in every case
  3 No need to inform at all

  - In what kind of cases is informing unnecessary?
    1 If the research topic is important for public health
    2 If the study is a continuation to a previous study of which I have already been informed about
    3 If a government research centre, a university, or some other reliable organization is in charge of the study
    4 If the amount of study participants is so high that informing all of them is extremely difficult
    5 Other. What? ____

- Would you like to be informed about the possible research use of your medical records when being admitted to hospital?
  1 No
  2 Yes
  3 Do not know

- Would you like to be able to limit or forbid the research use of your medical records?
  1 Yes, forbid altogether
  2 Yes, limit the use of some information
  3 No, everyone's information should be available for research
  4 Do not know

When conducting clinical intervention studies the research participants must be given information on the study and asked for a personal consent to take part in the study. In register-based research the number of the study participants is often very high and some of the people in the registers may be deceased. Obtaining informed consents from all the study participants for a register-based study would be extremely difficult or impossible. Under the current legislation in Finland, register-based research can be conducted without informed consents, if the study is based solely on register information. Instead of personal consents, the researchers apply for permission from a government official.

- In your opinion, should every research subject be asked for an informed consent for participating in a register-based study?
  1 No need for informed consent
  2 In some cases an informed consent should be obtained
  3 Informed consent should always be obtained

- In what kind of cases would you like to be asked for an informed consent?
    1 If the research topic or the information used for it are sensitive
    2 If the research results might stigmatize a group of people
    3 If the practical applicability of the study is unclear
    4 If the research topic is not of public health importance
    5 Other. What? ____

- What kind of policy would be best if informed consent was required for register-based research?
    1 Consent for every study
    2 One consent for a certain topic of research, for example cancer research
    3 One consent for the research use of a certain register
    4 Other. What? ____

Under the current legislation, people do not have the right to deny the research use of their register information. Conducting register-based research often requires combining information from different registers. Linking of register information is allowed without permission of the study subjects. Linkage is possible with social security number.

- In the table below there are different national registers. Would you like to be able to limit or forbid the research use of the information these registers contain?

	Yes, forbid the use altogether	Yes, limit the use of some information	No, everyone's information should be accessible for researchers	Do not know
Cancer register	1	2	3	4
Medical birth register	1	2	3	4
Care register for Health Care (HILMO)	1	2	3	4

- Should people have an option to limit or forbid linkage of different registers?
    1 Yes, to forbid linkage altogether
    2 Yes, to limit linkage of some registers
    3 No, information in every register should be available for linkage
    4 Do not know

Legislation

In other Scandinavian countries, the legislation involving register-based research is similar to Finland. The situation is different elsewhere in Europe. In Great Britain, record linkage is possible with a consent from the study participants. In Germany, combining the information in different registers is permitted by a data protection official.

- How do you feel about the differences in legislation concerning register-based research between European countries?
    1 I feel that the differences are not a problem
    2 I feel that the legislation in Europe should somehow be harmonized
    3 I feel that the legislation In Europe should be harmonized, closer to the Scandinavian model
    4 I feel that the legislation in Europe should be harmonized, the Scandinavian countries should shift closer to other European countries

- How would you like to change the Finnish legislation concerning register-based research?
    1 I would not change it at all. Current legislation is satisfactory.
    2 I would tighten it for some parts, for example linkage of different registers should be allowed only with a personal consent.
    3 I would tighten it significantly. The research use of national registers should be forbidden or only allowed with a special authorization and an informed consent.
    4 I would liberalize the laws. Conducting scientific research should be made as easy as possible.

Ethics Committee reviews

The ethical aspect of a clinical medical research is in Finland and many other countries evaluated by an Ethics Committee. The evaluation ensures that the study adheres to good scientific practice and that the study participants are being treated morally acceptably and that the benefits and possible harms form the study are balanced. In Finland the ethical evaluation is legally required only from studies that may cause physical harm to the study participants, leaving out register-based research.

- What is your opinion of Ethics Committee reviews for register-based research?
    1 Not needed for register-based research
    2 In some cases, for example if the research topic is sensitive, Ethics Committee review is needed.
    3 Should be required for every register-based study

- If the study was approved by an Ethic Committee, would your opinion about the need for informed consent change?
    1. No, informed consent is not needed.
    2. No, informed consent should anyway be obtained.
    3. Yes, in this case informed consent is no more needed.

- Do you have any concerns relating to register-based research?
    1. No concerns.
    2. Privacy protection, personal information ending up in wrong hands.
    3. The reliability of the researchers/research organizations is questionable.
    4. The importance of the research topics for public health is doubtful.
    5. The benefit for public health from register-based research is uncertain.
